# The Impact of the Scale of Third-Party Logistics Guaranteeing Firms on Bank Credit Willingness in Supply Chain Finance: An ERP Study

**DOI:** 10.3389/fpsyg.2022.853888

**Published:** 2022-04-12

**Authors:** Xuejiao Wang, Jie Zhao, Hongjun Zhang, Xuelian Tang

**Affiliations:** ^1^College of Science and Technology, Ningbo University, Ningbo, China; ^2^Institute of Neuromanagement, College of Science and Technology, Ningbo University, Ningbo, China; ^3^M.I.C.E and Tourism Development Research Base of Ningbo City, Ningbo, China; ^4^Department of Economics and Trade, Sejong University, Seoul, South Korea

**Keywords:** credit decision, third-party logistics guarantor financing, scale of guaranteeing firm, ERP, N2, LPP

## Abstract

Supply chain financing guaranteed by third-party logistics (3PL) firms is an effective way to solve the financing difficulties of small and medium-sized enterprises (SMEs). Studies have explored factors that affect the willingness of supply chain financial credit providers under guarantee of 3PL firms (e.g., the scale of financing enterprises and credit). However, whether the scale of 3PL firms will affect the bank’s credit decision has not been studied, as well as the neural processing of credit decisions. To clarify these issues, this study extracted behavioral and event-related potentials (ERPs) data when participants performed a selection task of judging whether to grant credit to guaranteed financing-seeking enterprises according to the large or small scale of the 3PL guaranteeing firms. The behavioral results showed that under the condition of a large-scale 3PL guaranteeing firm, the willingness to provide credit to SMEs was higher than that under the condition of a small-scale 3PL guaranteeing firm. This finding indicates there was credit scale discrimination against 3PL guaranteeing firms in supply chain finance. The ERP results showed that compared with the condition of a large-scale 3PL guaranteeing firm, a greater N2 amplitude was induced under the condition of a small-scale 3PL guaranteeing firm, which indicated that credit decision makers experienced greater perceived risk and more decision-making conflict. In contrast, a larger LPP amplitude was detected under the condition of a large-scale 3PL guaranteeing firm (as opposed to a small-scale firm), which indicated that large-scale 3PL guaranteeing firms received more positive comments and more positive emotions from credit decision makers than small-scale 3PL guaranteeing firms. Based on these results, this study reveals the cognition process of credit decision makers regarding the impact of the 3PL guaranteeing firm scale on the willingness to provide credit in supply chain finance and explains the theory of credit scale discrimination from the perspective of decision neuroscience.

## Introduction

Financial constraints are widespread worldwide. Compared with large companies, it is more difficult for small and medium-sized enterprises (SMEs) to obtain financing from banks and other financial institutions, and they are subject to greater financing constraints (Xavier, 2006; Ryan et al., 2013; Seo, 2013; Song et al., 2021). This difficulty not only severely restricts the development of such enterprises but also affects the income of other members of the supply chain and the supply chain overall. Guarantor financing is an effective way to resolve financing difficulties for SMEs (Randall and Farris, 2009; Martinez et al., 2020). Third-party logistics (3PL) guarantor financing has become one of the main methods to overcome such difficulties (Wang and Wang, 2019; Chakuu et al., 2020) by helping reduce banks’ risk perception and enhance their credit willingness. With the good credit of guaranteeing logistics firms’, SMEs in the same supply chain are more likely to obtain bank loans. That is, the risk of banks is greatly reduced in this situation, and they are more willing to lend.

To date, related studies on supply chain finance have mainly focused on innovation in the operation mode, benefit distribution mechanism, risk prevention (Caniato et al., 2016), and smart technology application (Rijanto, 2021) but have ignored the potential impact of the 3PL guaranteeing firm scale on credit willingness in supply chain finance. In reality, firm scale is an important factor that restricts various aspects of firm operation, which indicates its strength, capital, and future development prospects (Beck et al., 2005). Similarly, 3PL guaranteeing firm scale indicates that it is still solvent in the condition of a default by the guaranteed enterprise, which may have a significant impact on banks’ credit willingness in supply chain finance and thus requires further research. In the practice of supply chain finance, credit decision making is a complex process affected by many factors (Hofmann and Belin, 2011; Lekkakos and Serrano, 2016). Due to “information asymmetry, “the bank implements credit rationing and cannot meet the loan requirements of SMEs to avoid serious adverse selection (Joseph and Weiss, 1981; Kirschenmann, 2016). The application of smart technologies such as blockchain (Rijanto, 2021) and well-trained loan officers can minimize the impact of these factors. However, the supply chain finance credit is ultimately determined by the loan officer based on the materials submitted by the enterprise. Thus, this study focus on the decisions made by loan officers as an agency of bank decisions. It has been established that loan officers often pay attention to the scale of financing-seeking enterprises in supply chain finance when deciding whether to provide loans (Cenni et al., 2015; Ali, 2021). However, whether the scale of 3PL firms will affect the bank’s credit decision has not been studied, as well as the neurocognitive process and cognitive mechanism behind this behavior have not been fully studied. As a background against which to investigate these phenomena, this study examines how an enterprise seeking financing obtains bank credit under two conditions: when the guaranteeing firm is large-scale 3PL firm and when the guaranteeing firm is a small-scale 3PL firm.

To better to understand the impact of the scale of 3PL guaranteeing firms on banks’ credit willingness in supply chain finance, event-related potential (ERP) technology is applied (Plassmann et al., 2012; Pozharliev et al., 2015). ERPs are an important research method of decision-making neuroscience. The method offers the advantage of high time resolution, which can reflect different cognitive processes by observing the components affected by various experimental conditions through recording the potential on the scalp surface (Ma et al., 2012). A large number of neuroscience studies on decision making have shown the value of this approach to exploring potential conflict perception, emotional arousal, motivation, and stimulus evaluation in risk decisions, such as financial decision making (Frydman et al., 2014; Razi et al., 2016; Gianmario et al., 2017; Wuke et al., 2019). Thus, the method provides a foundation for us on which to conduct an ERP study on the impact of the scale of 3PL guaranteeing firm on the credit decision making of banks by revealing the cognitive processes that occur in the brains of loan officers. The findings of this study can provide insight into the implicit motivation of credit decision makers and serve as a supplement or explanation for results reported elsewhere (Ahlert et al., 2006; Yoon et al., 2006).

## Theoretical Background and Hypothesis Development

### Behavioral Hypothesis

Banks face various risks of the implementation of supply chain finance, such as supply risk, interruption risk, credit risk, market risk, and operational risk (Tsai, 2008; Shashank and Thomas, 2009; Chen et al., 2021). Due to the serious information asymmetry between banks and enterprises, banks implement credit rationing to SMEs to avoid risks (Xavier, 2006; Seo, 2013; Cenni et al., 2015). Adverse selection coming with the asymmetric information makes credit rationing a rational choice for banks (Stiglitz and Weiss, 1981). It has been widely believed that in the traditional Stiglitz–Weiss model, firm size plays an important role in influencing credit rationing (Wang et al., 2019). For example, some scholars conducted quantitative research using the data of Chinese listed companies from 2009 to 2015 and came to the following conclusions: Compared with large enterprises, SMEs obtained less credit financing and higher costs, that is, the phenomenon of credit scale discrimination does exist (Wei et al., 2019). This kind of situation that credit funds are tilted toward large-scale enterprises has seriously affected the growth of enterprises and their contribution to the economy (Beck et al., 2005). Some studies have found that the scale of financing enterprises in supply chain finance is an important factor affecting the implementation of supply chain finance (Ali, 2021). However, SMEs can enhance their image and reduce the information asymmetry between banks and such enterprises through the guarantee of 3PL firms (Randall and Farris, 2009; Bai, 2019). Thus, the existence of 3PL guaranteeing firms can effectively reduce the bank’s credit risk and increase its enthusiasm to support SME financing (Hofmann, 2005; Lamoureux and Evans, 2011; Wuttke et al., 2016). When a 3PL firm plays the role of guarantor in the supply chain finance of SMEs, to a certain extent, the scale of the 3PL guaranteeing firm represents in the view of the bank the firm’s ability to remain solvent in the event of a default (Deng et al., 2016). Therefore, the larger that the 3PL guaranteeing firm is, the smaller the bank’s perceived risk and the higher its willingness to grant credit; conversely, the lower its willingness to grant credit (Vousinas, 2018). Based on the preceding analysis, this article proposes the following hypothesis.

*H1*: The willingness of banks to provide credit to SMEs under the large-scale 3PL guaranteeing firm condition is higher than under the small-scale condition.

### ERP Hypotheses

Based on the described decision-making pattern, it is possible that the existence of 3PL guaranteeing firms reduces the perceived uncertainty and risk of credit personnel of financial institutions. Especially in the case of a large 3PL guaranteeing firm, the uncertainty and risk of credit perceived by financial institution personnel are low. Therefore, their evaluation of SMEs applying for loans is relatively high, which improves the credit willingness of the financial institution. This evaluation involves a value-based classification process. Thus, when studying the neural basis of the influence of the size of the guaranteeing 3PL firm on the credit decision making of financial institutions in supply chain finance, this study focuses on two ERP components that are often studied in decision-making research and are closely related to decisional conflict and risk perception (i.e., N2) and evaluation classification (i.e., LPP).

### N2

The N2 component is a negative potential that peaks at 200 and 400 ms after stimulation. It is mainly distributed among the frontal area, frontal central joint area, and central area of the brain. It is an ERP component often studied in decision-making research (Nagy et al., 2003; Handy, 2005; Wuke et al., 2019). A considerable number of studies had consistently suggested that N2 is a conflict-related component and whose amplitude is positively correlated with decisional conflict (Yang et al., 2007; Larson et al., 2012; Han et al., 2015; Wang et al., 2018; Yu et al., 2020). Recently, studies have begun to show that when experimental subjects are faced with information flows from different sources, their perceived conflicts of decision-making risks are reflected by the amplitude of the N2 component (Ma et al., 2015; Meng and Xiu, 2018), which indicates that higher perceived risk attracts greater attention resources and increases the difficulty of decision making (Wang et al., 2018; Wuke et al., 2019). For example, the N2 amplitude in the decision making of high-risk PPP financing projects is significantly higher than that of low-risk projects, and the N2 amplitude increases with the increase in conflicts in risk management decisions (Meng and Xiu, 2018).

Generally, the 3PL firms that provide guarantees of supply chain finance is relatively stronger and larger enterprises (Abbasi et al., 2017; Wang and Wang, 2019). Firm size has a positive relationship of supply chain finance practices (Uyar, 2009; Ali, 2021). It has been established that loan officers often pay attention to the scale of financing-seeking enterprises and 3PL guarantor firms in determining whether to provide loans (Cenni et al., 2015; Ali, 2021). We have speculated in the behavioral hypothesis that the loan officers are likely to believe that under the guarantee of the large-scale 3PL guaranteeing firm, it is a good choice to grant credit to financing-seeking enterprises in supply chain finance. Conversely, while the 3PL guaranteeing firm is small-scale, loan officers are likely to believe that granting credit to the financing-seeking enterprises is accompanied by more uncertainty and risk, and cannot assure it is a good choice. In other words, the size of 3PL guaranteeing firms is directly related to the risk perception of credit decision makers. Higher risk perception will attract more attention resources and increase the difficulty of decision making, it will cause a larger N2 amplitude (Wang et al., 2018; Wuke et al., 2019). Specifically, we hypothesize the following.

*H2*: The N2 amplitude of the supply chain financial credit decision under the conditions of the participation of a large-scale 3PL guaranteeing firm will be smaller than under the condition of a small-scale guaranteeing firm.

### LPP

LPP (late positive potential) is a late positive-going component that mainly distributes over the central-parietal regions and peaks at approximately 600 ms after the onset of stimuli (Herring et al., 2011). Previous studies consistently show that the magnitude of LPP reflects the allocation of attention resources. The more attention resources that subjects obtain, the greater the amplitude of LPP (Dolcos, and F., 2006). Studies on decisions have recently reported that LPP may reflect the cognitive process of assessment classification (Chen et al., 2010; Wang et al., 2016; Wuke et al., 2019). These studies suggest that an increase in LPP amplitude is associated with better evaluation and classification of stimuli (Chen et al., 2010; Wang et al., 2016; Wuke et al., 2019). For example, product descriptions that imply lower risk and better future performance lead to a greater amplitude of LPP (Wang et al., 2016; Wuke et al., 2019).

Studies have shown that banks make credit decisions based on the group characteristics of the credited companies (Riley, 1987; Brandt and Li, 2002). In practice, 3PL guaranteeing firms in supply chain finance are all large-scale companies. Banks believe that large 3PL guaranteeing firms have advantages in terms of information acquisition, industry experience, and solvency (Zhou et al., 2020; Hua et al., 2021). Therefore, it is reasonable to say banks evaluate a large 3PL guaranteeing firm better than a small one. In the current study, we assume that banks granting credit to a large-scale 3PL guarantee financing enterprise is with less uncertainty and risk than those granting credit to a small 3PL guarantee financing enterprise. As increased LPP amplitude is positively related to higher evaluation categorization (Wang et al., 2016; Wuke et al., 2019), we speculate that financing-seeking enterprises guaranteed by a large 3PL guaranteeing firm, rather than a small 3PL guaranteeing firm, will lead to a better evaluation, which will be reflected in a greater LPP amplitude. Thus, the following hypothesis is proposed.

*H3*: The amplitude of LPP under the condition of the participation of a large-scale 3PL guaranteeing firm will be greater than under the condition of a small-scale 3PL guaranteeing firm.

## Materials and Methods

### Participants

The experiment recruited 30 Chinese students (16 females) from senior or graduate students majoring in finance-related subjects in Ningbo University, aged 19–28 years (*M* = 20.53 years, SD = 2.177 years) as paid volunteers. All participants were native Chinese speakers, right-handed, and had normal or corrected vision without a history of psychological or mental illness. Prior to the experiment, all participants were informed of the experimental procedures and other relevant details and signed a written informed consent form, indicating their voluntary participation in the experiment. The research was conducted in accordance with the Declaration of Helsinki (WMA, 2013). After the experiment, the participants were paid 40 RMB. In the formal experiment, the data of four subjects were discarded due to excessive ERP artifacts. Therefore, the final analysis included 26 active participants. The experiment was approved by the Ethics Committee of the School of Neuroeconomics and Neuromanagement of Ningbo University.

### Stimulus Materials and Stimulus Selection

This research investigates 3PL firms of different sizes that provide financing guarantees on behalf of SMEs. The study examines two loan decision-making situations (large vs. small 3PL guaranteeing firm).

To determine the scale of the guaranteeing logistics firms, a questionnaire survey was conducted on the names of the guaranteeing logistics firms investigated in the experiment, the 30 participants (16 women) were asked to rate 50 companies with the same name length using a 5-point Likert score (1 = minimum scale; 5 = maximum scale). Ten small 3PL guaranteeing firms with an average scale of less than 2.53 points and 10 large 3PL guaranteeing firms with an average scale of greater than 4.55 points were selected. Subsequently, the same method was used to select financing enterprises of different sizes as stimulus materials. Thus, in the formal experiment, 8 financing-seeking enterprises and 20 3PL guaranteeing firms (10 large and 10 small) were combined to create 160 trials. The stimulus material was displayed in the middle of a gray screen of a size of 270*360 pixels. The 28 displayed company names were the same in length, with a font of 30 points, and corresponding symbols were used to indicate the size of the company under the company name. The symbols were used for both the financing-seeking companies and the 3PL guaranteeing firms (i.e., 1 or 2 ☆ = small; 4 or 5 ☆ = large). All stimuli were randomly divided into 4 blocks, each with 40 trials. In each block, 40 trials were performed in pseudorandom order.

### Procedures

First, participants were informed that they must observe the names and scales of the financing-seeking companies and 3PL guaranteeing firms in turn. Next, they were to imagine that they, as credit decision makers, were determining whether they were willing to grant credit to each financing-seeking company and to score their willingness to provide credit on a continuum from 1 (very reluctant) to 7 (very willing). The participant was seated comfortably in a sound-reduced room, approximately 100 cm from a computer-controlled monitor (1,280 × 1,024 pixels) with a refresh rate of 60 Hz, the stimulus material was displayed in the middle of the screen. The participants were instructed to make their decisions using a wireless keyboard.

**Figure 1:**
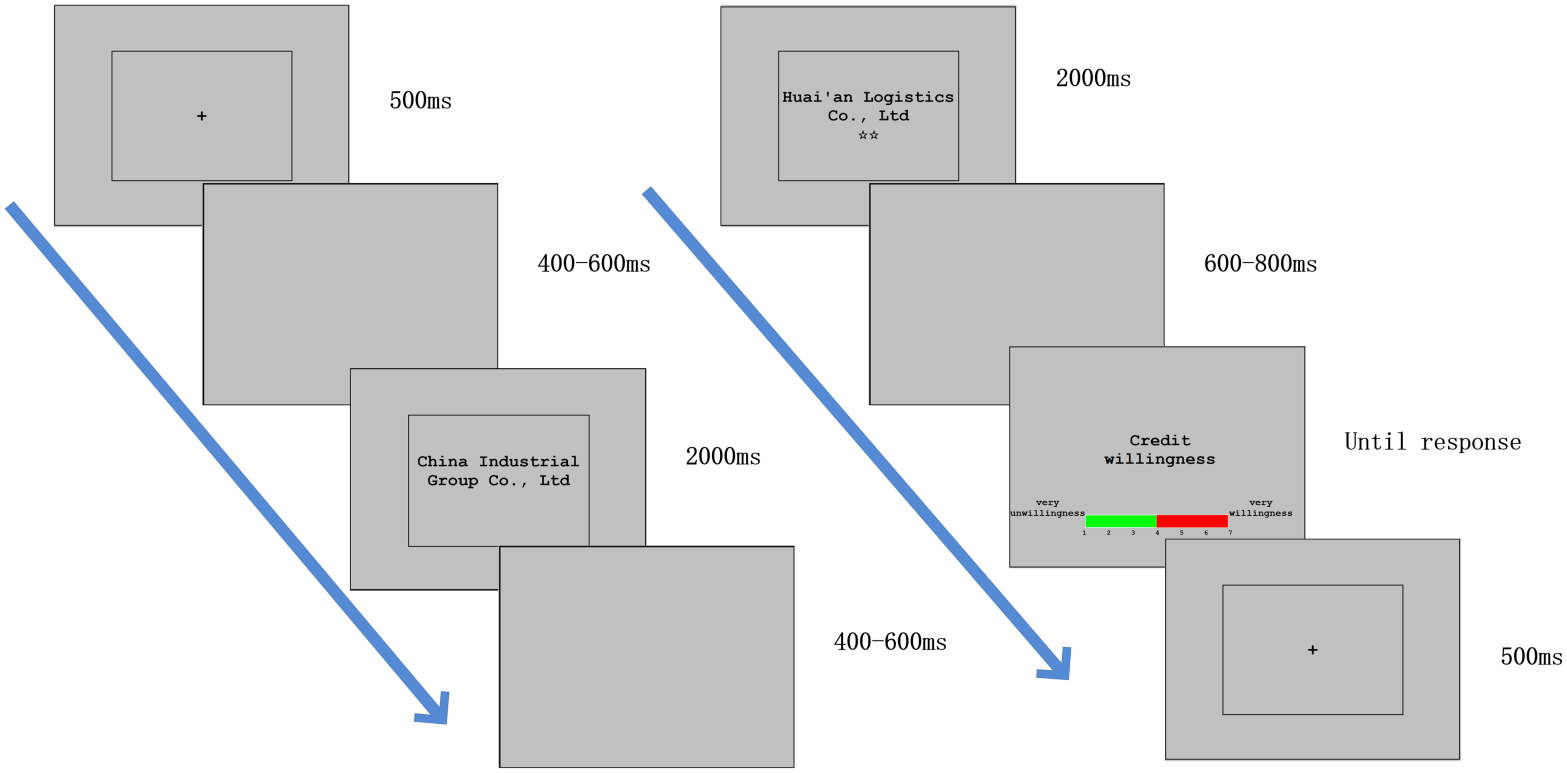
Experimental task: Participants were instructed to determine whether to grant credit based on the scale of 3PL guaranteeing firms.

As shown in Figure 1, at the beginning of each test, a fixed cross appeared for 500 ms, followed by a blank screen that appeared for 400–600 ms. Then, the name of the financing-seeking enterprise (with a fixed length) appeared for 2,000 ms, followed by a blank screen that appeared for 600–800 ms. Next, the name of a 3PL guaranteeing firm (fixed length; 2,000 ms) and the number of stars representing its scale were displayed, followed by a blank screen that appeared for 600–800 ms. Finally, the participants were asked to rate their intentions of granting credit to the financing-seeking enterprise according to the scale of the 3PL guaranteeing firm. The triggers for stimulation and recording were presented using the E-Prime 2.0 software package (Psychology Software Tools, Pittsburgh, PA, United States). The participants were also asked to minimize blinking, eye movement, and muscle movement during the experiment. The formal experiment commenced after 6 practice trials.

### Electroencephalogram Recordings and Data Analysis

EEG recording involves the use of a cap with 64 Ag/AgCl electrodes whose potential distribution is in accordance with the international 10–20 system standard. The sampling frequency was set to 500 Hz, the online bandpass filter frequency was set to an eego amplifier of 0.1–100 Hz (all produced by ANTNeuro, Enschede, Netherlands). A pair of electrodes placed 10 mm above and below the right eye were used to record the vertical electrooculograms, while another pair of electrodes placed 10 mm to the right of the right eye and 10 mm to the left of the left eye were used to record the horizontal electrooculograms. The forehead position was used as the ground, and the left mastoid was used as a reference. The impedance of the tested scalp was maintained below 5 kΩ to ensure good data quality.

The left mastoid reference was transferred to the arithmetic mean of the bilateral mastoid as the offline reference. The method proposed by Semlitsch was used for ocular artifact correction (Semlitsch et al., 2010). A 30 Hz (24 dB/Octave) low-pass filter was used to digitally filter the average ERP. The 200 ms prior to the onset of stimulus (i.e., the name and scale of the financing-seeking company or the guaranteeing company) and the 800 ms after this onset were used as the epochs, and the 200 ms prior to the onset of stimuli was used as the baseline. The data collected after the stimulus presentation were compared with the corrected baseline value. EEG data whose amplitude exceeded ±100 μm were removed to improve the quality of the statistical data.

This study adopted a within-subjects experimental design; therefore, repeated-measures ANOVA within the subjects was used for the data statistics, Greenhouse–Geisser correction (Greenhouse and Geisser, 1959) and Bonferroni correction were used for correction according to the experimental conditions, and partial eta-squared values ( $\eta_{p}^{2}$ ) are reported to demonstrate the effect sizes in the ANOVA models (Cohen, 1988). Based on previous research and visual observation of the scalp distribution of the large average waveform in our research, 9 electrodes (F1, Fz, F2, FC1, FCz, FC2, C1, Cz, and C2) were selected for N2 component analysis, and another 9 electrodes (C1, Cz, C2, CP1, CPz, CP2, P1, Pz, and P2) were used for LPP component analysis. In addition, the time windows for responding to the N2 and LPP components were 190–240 ms and 400–550 ms, respectively. Then, a 2 (3PL guaranteeing firms: large vs. small) × 9 (electrodes: F1, Fz, F2, FC1, FCz, FC2, C1, Cz, and C2) ANOVA was conducted for the mean amplitude of the N2 component, while a 2 (3PL guaranteeing firms: large vs. small) × 9 (electrodes: C1, Cz, C2, CP1, CPz, CP2, P1, Pz, and P2) ANOVA was performed for the mean amplitude of the LPP component.

## Results

### Behavioral Results

According to the research hypotheses, statistical analysis was performed on the credit willingness of the 26 subjects. The *t*-test results of the paired samples show that the main effect of the difference in the willingness to provide credit of the financial institutions under the conditions of 3PL guaranteeing firms of different sizes is significant, and the participants displayed higher willingness to grant credit under the condition of a large-scale 3PL guaranteeing firm [M_big_ = 5.5067, SE = 0.128; M_small_ = 3.1804, SE = 0.104; *t*(1,25) = 19.145, *p* = 0.000]. Therefore, H1 is supported.

### ERP Results

#### N200 (190–240 ms)

**Figure 2:**
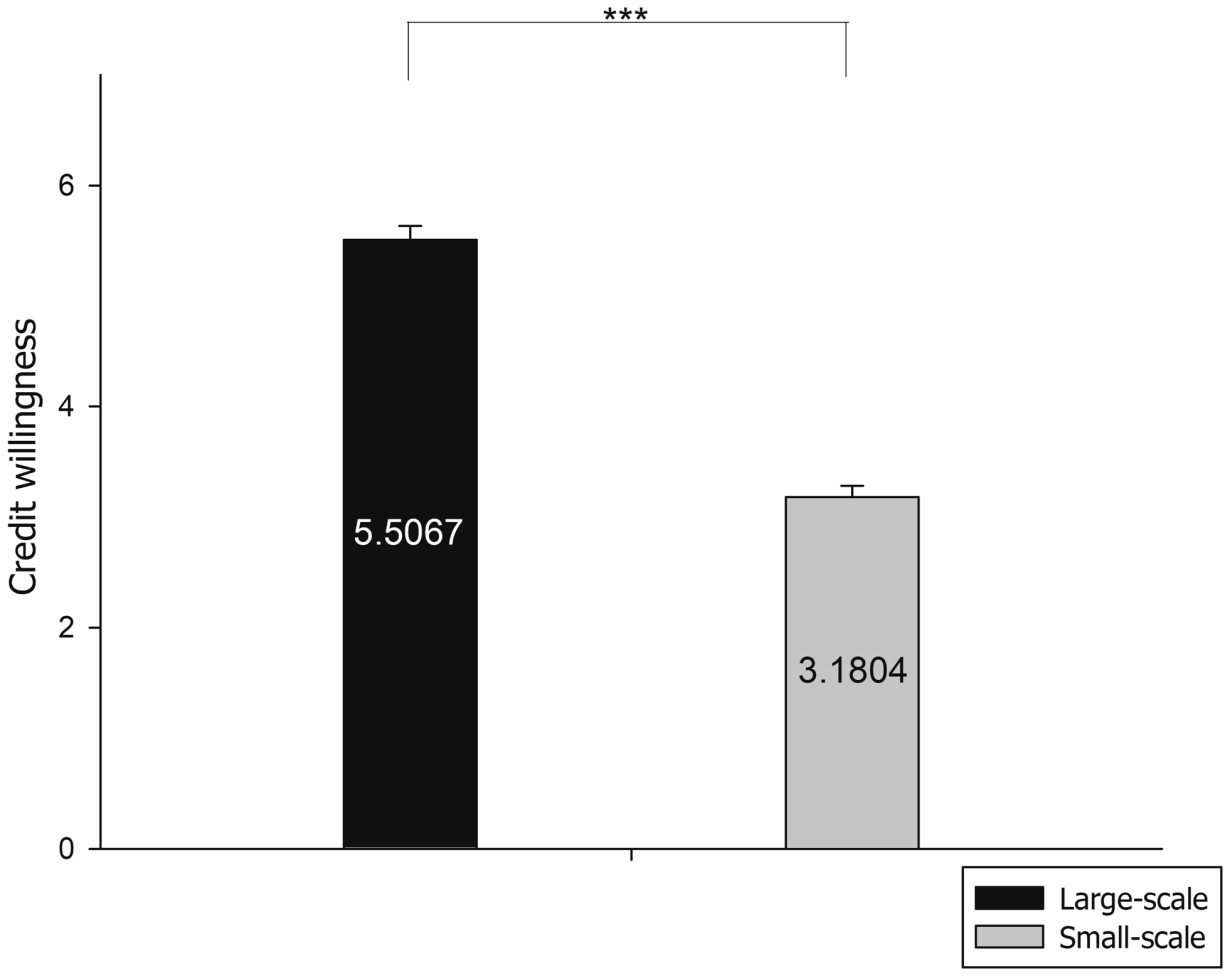
Behavioral results for the willingness to provide credit (large-scale vs. small-scale). ***p<0.001.

We conducted a two-way 2 (3PL guaranteeing firms: large vs. small) × 9 (electrodes: F1, Fz, F2, FC1, FCz, FC2, C1, Cz, and C2) repeated measure ANOVA for the mean N2 amplitude. The results reveal that the main effect of different scale 3PL guaranteeing firms is significant [*F*(1, 25) = 26.796, *p* = 0.000, $\eta_{p}^{2}$ = 0.517], which indicates that the average amplitude (negative polarity: when the voltage value is small, the amplitude is larger) under the condition of a small-scale 3PL guaranteeing firm (M_small_ = 0.138 μV, SE = 0.579) is larger than under the condition of a large-scale 3PL guaranteeing firm (M_big_ = 1.636 μV, SE = 0.524; Figure 2).

**Figure 3:**
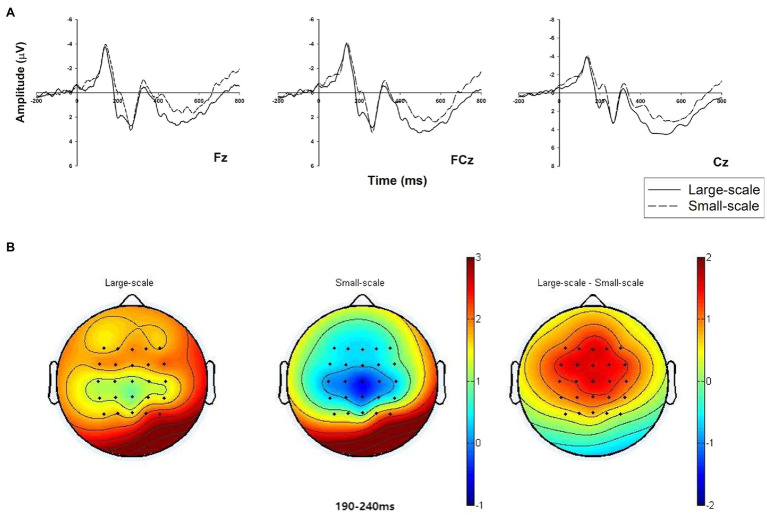
Grand-averaged event-related potential (ERP) waveforms of N2 in the frontal-to-central region with three electrodes and related brain topography. **(A)** The N2 amplitude comparison under the two conditions of 3PL guaranteeing firms (large-scale vs. small-scale) at representative electrodes (Fz, FCz, and Cz). **(B)** Brain topography of the two conditions and a contrast at the N2 time window of 190–240 ms.

The N2 analysis results support H2. We select three midline electrodes (Fz, FCz, and Cz) to illustrate their neural dynamic activity under the conditions of different sizes of 3PL guaranteeing firm (Figure 3A). In addition, the brain map shows the main differences between the two conditions from the frontal lobe to the central region (Figure 3B).

#### LPP (400–550 ms)

We conducted a two-way 2 (3PL guaranteeing firms: large vs. small) × 9 (electrodes: C1, Cz, C2, CP1, CPz, CP2, P1, Pz, and P2) repeated measure ANOVA for the mean LPP amplitudes. The results revealed that the main effect is significant [*f*(1,25) = 9.197, *p* = 0.006, $\eta_{p}^{2}$ = 0.269], which indicates that the average amplitude of LPP under the condition of a large-scale 3PL guaranteeing firm (M_big_ = 5.375 μV, SE = 0.677) is larger than under the condition of a small-scale 3PL guaranteeing firm (M_small_ = 3.921 μV, SE = 0.756; Figure 3).

**Figure 4:**
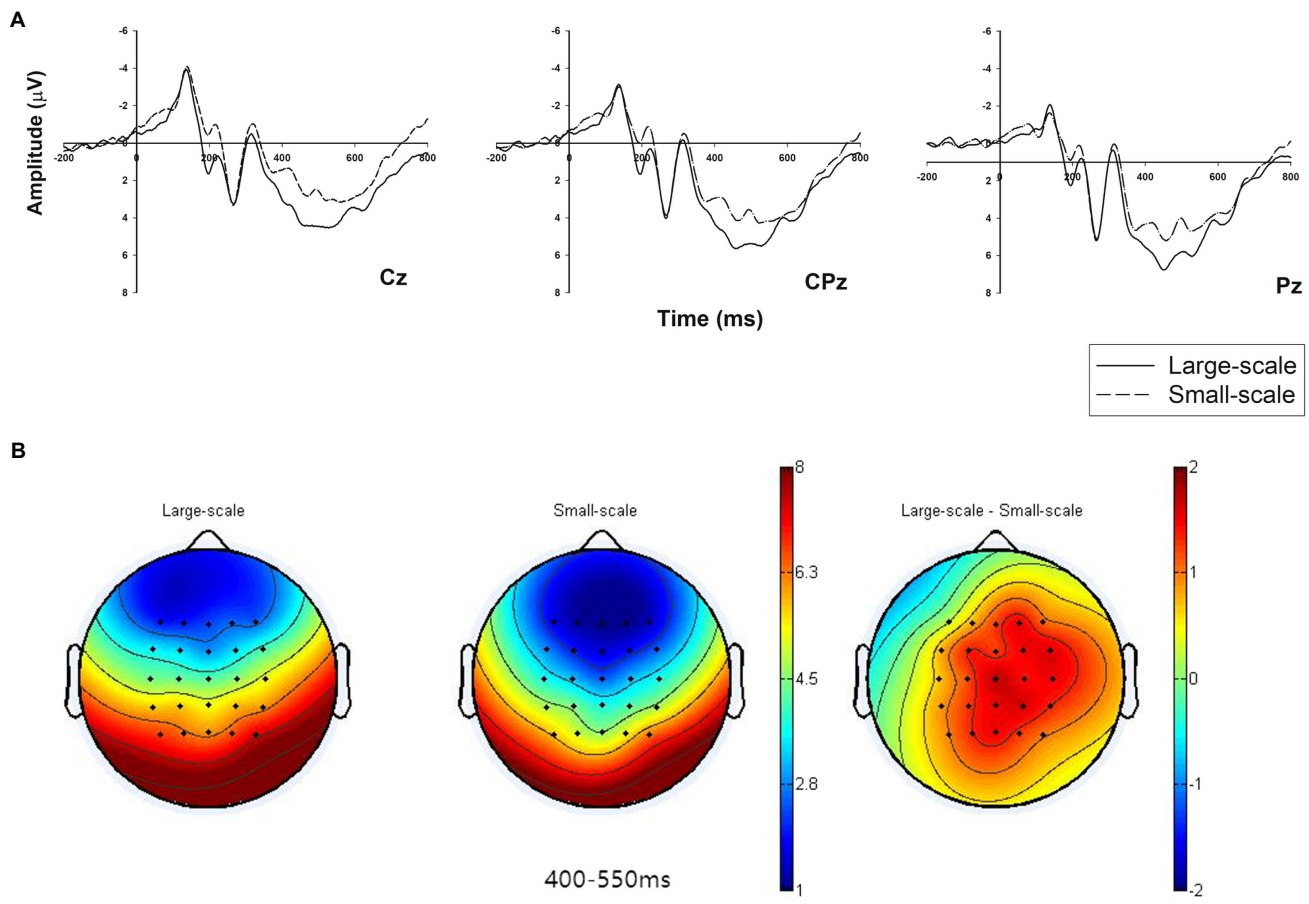
Grand-averaged event-related potential (ERP) waveforms of LPP in the central-to-parietal region with three electrodes and related brain topography. **(A)** The LPP amplitude comparison of the two conditions of 3PL guaranteeing firms (large-scale vs. small-scale) at representative electrodes (Cz, CPz, and Pz). **(B)** The brain topography of the two conditions and a contrast at the LPP time window of 400–550 ms.

The LPP result supports H3. Three intermediate electrodes (Cz, CPz, and Pz) were selected, and their neural dynamic activity under the conditions of different sizes of 3PL guaranteeing firm is illustrated in Figure 4A. In addition, the brain topographic map shows the main differences between the two conditions in the central-to-parietal region (Figure 4B).

#### Correlation Analysis Between EEG Data and Behavior

**Figure 5:**
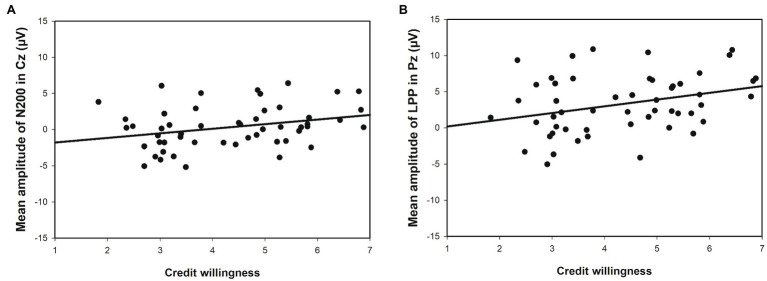
Correlation between amplitudes of N2 or LPP and credit willingness: **(A)** Linear correlation between the mean amplitude of N2 at the Cz electrode and credit willingness; **(B)** Mean amplitude of LPP at the Pz electrode and credit willingness.

A correlation analysis of the average amplitudes of N2 and the decision-making behavior revealed a significant positive correlation between the average amplitudes of N2 at the Cz electrode and the behavior (*r* = 0.306, *p* < 0.05; Figure 5A). In addition, we analyzed the correlation between decision-making behavior and the average amplitude of LPP. The results revealed a significant positive correlation between the average amplitude of LPP at the Pz electrode and behavior (*r* = 0.353, *p* < 0.05; Figure 5B).

## Discussion

### Key Findings

This study used ERPs to investigate the influence of the size of a 3PL guaranteeing firm on bank credit decision making in supply chain finance and its potential neural mechanism. To the best of our knowledge, this study is the first to apply the neuroscience method of ERPs to study credit decision making in supply chain finance. Significant effects were observed at both the behavioral and neural levels. First, the behavioral results showed that loan officers had a higher willingness to grant credit under the condition of a large-scale 3PL guaranteeing firm than under the condition of a small-scale firm. Regarding response time, there was no significant difference between large 3PL guaranteeing firm and small firm. The ERP results showed that supply chain financial decision making when a large 3PL guaranteeing firm is involved results in a smaller N2 amplitude. This outcome indicates that credit decision makers perceive a relatively low risk and thus reduced conflict and difficulty in decision making. In addition, the participation of a large 3PL guaranteeing firm in supply chain finance results in a larger average amplitude of LPP than a small guaranteeing firm participates. This outcome indicates that the involvement of a large 3PL guaranteeing firm leads to higher positive evaluation.

### Behavior Result: Credit Scale Discrimination Occurs in Supply Chain Financial Credit Decision Making

The behavioral results reveal that credit decision makers have a higher willingness to provide credit under the condition that the 3PL guaranteeing firm is large. It is found that banks discriminate against 3PL firms that offer guarantees for SME financing enterprises. Previous studies have shown that under the condition of incomplete information, it is difficult for banks to monitor the possible default motives of SMEs (Ray, 1998), resulting in scale discrimination in SME credit financing (Gertler and Gilchrist, 1994; Kudlyak and Sánchez, 2016). Behavioral finance theory holds that information asymmetry causes banks to have cognitive biases on financing companies, leading to biased credit decision making (credit rationing; Joseph and Weiss, 1981; Stiglitz and Weiss, 1981). Some studies have proved that collateral (i.e., “hard information”) and corporate reputation (i.e., “soft information”) can effectively alleviate the problem of information asymmetry in credit rationing (Bester, 1987; Diamond, 1989). However, SMEs have few collaterals, lack effective “hard information,” and it is difficult to obtain “soft information” such as high corporate reputation. The characteristics of risk bundling and information sharing in supply chain finance can reduce the degree of information asymmetry between banks and SMEs and greatly reduce the risk and monitoring cost of commercial banks providing loan services for such enterprises (Yang et al., 2021). However, in certain cases, banks still face the risk caused by SMEs, which reduce the willingness of banks to perform supply chain financing. In this study, although we provided credit decision makers with information on the 3PL firms offering guarantees for the credit-seeking SMEs, these decision makers remained unsure whether credit for the SMEs was the right choice. After all, the scale of 3PL guaranteeing firms indicates solvency in the event of a default by the guaranteed SME. Large-scale 3PL guaranteeing firms have strong solvency which can reduce the risk of supply chain finance. And small-scale 3PL guaranteeing firms do not have the corresponding debt-paying ability which cannot reduce the risks brought by SMEs in supply chain finance. Thus, the scale of 3PL guaranteeing firms has a significant impact on banks’ willingness to grant credit in supply chain finance. Credit decision makers may (in part) make decisions based on the scale of the 3PL firms that provide guarantees for SMEs. The scale of the 3PL guaranteeing firm is small, bankers may think that granting an SME credit is a dangerous choice and not conducive to increasing the bank’s income, they refuse to take the risk. In contrast, if a large 3PL firm offers to guarantee, the bankers may think that credit for the SME is a good choice.

### Neural Mechanism: Decisional Conflicts and Evaluation Classifications Exist in the Supply Chain Financial Credit Decision Process

The results of event-related potential revealed that large-scale 3PL guaranteeing firms induce smaller N2 amplitudes and larger LPP amplitudes than small-scale firms. These results are explained as follows. Banks are more inclined to provide credit to SMEs affiliated with large, well-developed and leading 3PL logistics firms (Vousinas, 2018). Credit decision makers believe that enterprise size has a positive relationship of supply chain finance practices (Uyar, 2009; Ali, 2021). In contrast, they tend to think it is dangerous to provide financing to enterprises affiliated with small-scale 3PL guaranteeing firms. The higher perceived risk of the decision-making processes increases the difficulty of decision making and further aggravates decision-making conflict (Wang et al., 2018; Wuke et al., 2019). Under the condition of a small-scale 3PL guaranteeing firm, decision conflict is greater, the N2 amplitude is greater. Therefore, a small 3PL guaranteeing firms lead to higher perceived risk, which explains the theory of credit scale discrimination from a new perspective.

We also find that the participation of large-scale 3PL guaranteeing firms in supply chain finance results in a greater LPP amplitude than that of small-scale firms. A large number of studies have found that LPP components may reflect the neurophysiological mechanism of classification processing (Chen et al., 2010; Wang et al., 2016; Wuke et al., 2019). These studies suggest that an increase in LPP amplitude is associated with better evaluation of classified stimuli (Chen et al., 2010; Wang et al., 2016; Wuke et al., 2019). Many researchers believe that enterprise size is positively related to the proportion of bank credit in total assets (Rajan and Zingales, 1998). That is, the larger that the scale of an enterprise is, the better its access to bank credit. The participants in this study may have more highly evaluated the financing-seeking enterprises guaranteed by large-scale 3PL firms and classified them in better evaluation categories (compared with small-scale 3PL guaranteeing firms), reflecting the larger amplitude of LPP. Thus, large 3PL guaranteeing firms obtained more positive comments and more positive emotions from the credit decision makers.

### Theoretical Contribution and Implications for Practice

The findings of this study have significance for industry practitioners. First, it is the first attempt to study supply chain finance from the perspective of cognitive neuroscience, the study reveals that scale discrimination occurs in supply chain financial credit decision making with respect to 3PL guaranteeing firms. Many scholars believe that guarantor financing is an effective way to solve the financing problems of SMEs (Randall and Farris, 2009). However, this study found that banks discriminate against 3PL firms that guaranteeing for SME financing enterprises. Thus, the credit willingness of bank is reduced. Therefore, supply chain finance does not proceed smoothly in practice not because there is no guarantor but likely because there is no strong guaranteeing firm to reduce the information asymmetry between banks and the financing-seeking enterprises. Furthermore, participants did not bear the loss of high risk of the selected study, choosing the high-risk option may be more prominent than in the real world (Nieuwenhuis, 2011). Based on the findings of this study, we should further to examine the causes of such discrimination in supply chain finance to avoid the decision-making bias of credit decision makers as much as possible. Second, neurocognitive tools can be applied for financial decision making. Credit decision makers occasionally cannot explain why they exhibit specific behavior (Dijksterhuis, 2004; Berns and Moore, 2012; Plassmann et al., 2015) or do not know their own thoughts and feelings (Chamberlain and Broderick, 2007; Thayer, 2010; Pozharliev et al., 2015). In this study, decisional conflicts and evaluation classifications exist in the supply chain financial credit decision process. 3PL guaranteeing firm affects the credit decision makers’ perception matching degree and subsequently affects a series of psychological processes, such as the attention to enterprise scale and emotional attitude, finally affecting their credit decision making regarding guaranteed enterprises seeking financing. This paper reveals the cognition of credit decision makers regarding the scale of 3PL guaranteeing firms and the psychological mechanism of credit decision making for guaranteed financing-seeking enterprises, which can provide insight into the implicit motivation of credit decision makers and serve as a supplement or explanation for results reported elsewhere.

### Limitations and Future Research

There are some limitations that should be acknowledged. First, there are limitations in the research sample. In this study, college students were selected to participate in the experiment, considering the control of individual factors, experimental efficiency, experimental cost control, and the convenience of data acquisition. Because the selection of this kind of experimental object can well control the interference factors, which is conducive to explore the general law. However, it will have more extensive value if we can take the real loan officer as the research object. The professional knowledge, bank, and business environment of the credit decision maker may affect the credit decision making. Therefore, if we can recruit real bank credit officers as participants, it will enhance the comprehensive understanding of supply chain financial credit decision making and the generalization of research conclusions. Second, other decision types in supply chain finance are also not considered in this study. Supply chain financial decision making may involve many complex problems (Rijanto, 2021), which requires professionally trained loan officers to support decision making. Therefore, it is not clear whether the loan officers’ credit decision-making choices specifically refer to their business expertise. Third, it is possible that the ERP components related to the neural mechanism behind the credit decision of supply chain finance have not been fully discovered. Some studies have examined P3 ERP components and early error detection ERP components (FRN, ERN, and MFN) elicited following risk-related decisions or task feedback (Dilushi et al., 2018). However, these components were not found in this study, which may be related to specific decision-making situations, and future research needs to further explore. SMEs credit institutions and loan officers should be fully aware of their limitations. On the whole, one should be careful when generalizing the conclusions of this work to larger populations or real-world decisions.

## Data Availability Statement

The raw data supporting the conclusions of this article will be made available by the authors, without undue reservation.

## Ethics Statement

The studies involving human participants were reviewed and approved by the Ethics Committee of the Academy of Neuroeconomics and Neuromanagement at Ningbo University. The patients/participants provided their written informed consent to participate in this study.

## Author Contributions

XW made substantial contributions to the research, participated in all aspects of manuscript production, conducted experiments, analyzed the data, and wrote the manuscript. JZ and HZ made significant contributions to the research and participated in all aspects of manuscript production. XT participated in data acquisition and data interpretation. A quality management company was responsible for supervising the study and managing each of its parts. All authors contributed to the article and approved the submitted version.

## Funding

This research was supported by Project of Ningbo M.I.C.E and Tourism Development Research Base: Research on the construction of the Ningbo national free trade zone and the coordinated development of tourism (JD5-PY33) as well as the Humanities and Social Sciences Cultivation Project of Ningbo University: Research on the Mechanism and Path of the Digital Transformation of Supply Chain in Zhejiang Province (XPYB20007). And it was supported by soft science project of Zhejiang Province (2021C35057): Research on Cross-border Innovation Path and Support Policy Suggestion of Zhejiang Service-oriented Enterprises in the Post Epidemic Era.

## Conflict of Interest

The authors declare that the research was conducted in the absence of any commercial or financial relationships that could be construed as a potential conflict of interest.

## Publisher’s Note

All claims expressed in this article are solely those of the authors and do not necessarily represent those of their affiliated organizations, or those of the publisher, the editors and the reviewers. Any product that may be evaluated in this article, or claim that may be made by its manufacturer, is not guaranteed or endorsed by the publisher.
